# Comparative Effects of Antimicrobial Stewardship Interventions on Pediatric Antibiotic Prescribing: A Systematic Review of Randomized Controlled Trials

**DOI:** 10.7759/cureus.109648

**Published:** 2026-05-25

**Authors:** Abdulelah F Alshehri, Nasser Saad Alshaiban, Asaad H Alidarous, Rana Alhazzani, Mohammad Majrashi, Saad Khalid AlMugbel, Ahmed S Alzailaei, Meshari Dalbouh, Salman Ali M Asiri, Mousa Naser M Asery, Yaser S Alharthi

**Affiliations:** 1 College of Medicine, Imam Mohammad Ibn Saud Islamic University (IMSIU), Riyadh, SAU; 2 Department of Pediatrics, King Saud University Medical City, Riyadh, SAU; 3 Department of Pediatric Emergency, Hera General Hospital, Makkah, SAU; 4 Epidemiology, Independent Researcher, Riyadh, SAU; 5 Department of Neonatology, Al Yamamah Hospital, Riyadh, SAU; 6 Department of Pediatric Neurology, King Saud University Medical City, Riyadh, SAU; 7 Department of Pediatric Emergency, Khamis Mushait Maternity and Children Hospital, Khamis Mushait, SAU; 8 Pediatric Emergency Medicine, King Fasial Medical City, Abha, SAU; 9 Department of Pediatric Emergency Medicine, Armed Forces Hospital, Khamis Mushait , SAU; 10 Department of Pediatric Emergency Medicine, Maternity and Children's Hospital, Abha, SAU

**Keywords:** antibiotic overuse, antimicrobial stewardship, appropriate antibiotic prescribing, clinical decision support, primary medical care

## Abstract

Acute respiratory tract infections (ARTIs) are a major driver of antibiotic prescribing in pediatric outpatient care, much of which is inappropriate. Multiple randomized trials have evaluated antimicrobial stewardship interventions; however, the consistency of effects across different intervention mechanisms remains unclear. We conducted a systematic review of randomized and cluster-randomized controlled trials evaluating interventions aimed at reducing antibiotic prescribing for pediatric ARTIs in outpatient and primary care settings. Searches were performed in PubMed, Embase, the Cochrane Library, and Google Scholar from inception to January 2026. Due to substantial clinical and methodological heterogeneity, findings were synthesized narratively. Eight randomized controlled trials were included. Intervention effects were heterogeneous, with strategies incorporating accountability and performance feedback demonstrating more consistent directional effects toward reduced or more appropriate antibiotic use. In contrast, shared decision-making, communication-based, diagnostic-guided, and stand-alone digital interventions showed mixed or neutral effects. Where reported, reductions or neutral effects were not associated with increased reconsultation, hospital admission, or symptom deterioration. Interventions incorporating accountability mechanisms demonstrated more consistent influence on pediatric antibiotic prescribing than single-component strategies, supporting the use of multicomponent antimicrobial stewardship approaches in outpatient pediatric care.

## Introduction and background

Acute respiratory tract infections (ARTIs) remain the most frequent cause of pediatric consultations in primary care and outpatient settings worldwide. Although most episodes are viral and self-limiting, antibiotics continue to be prescribed at high rates, making respiratory diagnoses the leading driver of outpatient pediatric antibiotic use [1]. Large population-based studies consistently show that a substantial proportion of these prescriptions are inappropriate or discordant with guideline recommendations [2]. Beyond antimicrobial resistance, inappropriate antibiotic exposure in children is now recognized as a patient-safety issue, being associated with avoidable adverse drug events, excess healthcare utilization, and increased costs [3]. Recent multinational surveillance further demonstrates that even when antibiotics are clinically indicated, suboptimal drug selection and duration remain common in ambulatory pediatric care, indicating persistent quality gaps [4].

The determinants of inappropriate prescribing for pediatric ARTIs extend beyond clinical knowledge alone. Diagnostic uncertainty, time pressure, clinician risk aversion, and perceived caregiver expectations interact to promote precautionary prescribing at the point of care [5]. Behavioral and implementation science frameworks suggest that durable changes in prescribing behavior are more likely when interventions address multiple domains of influence, including clinician capability, environmental opportunity, and motivational drivers. Consequently, single-component educational strategies may be insufficient to produce consistent effects across heterogeneous outpatient settings. Antimicrobial stewardship interventions aim to optimize antibiotic prescribing by improving appropriateness, reducing unnecessary exposure, and supporting evidence-based clinical decision-making. Over the past decade, numerous randomized controlled trials have evaluated outpatient pediatric stewardship interventions using pragmatic and cluster-randomized designs. These interventions can be conceptually grouped into four mechanistic categories: educational and stewardship-based approaches, behavioral and shared decision-making strategies, diagnostic-guided tools, and digital clinical decision support systems, which provide point-of-care prescribing guidance [6]. Evidence from randomized trials suggests that intervention effectiveness varies according to mechanism and implementation context. Background outpatient antimicrobial stewardship evidence, including audit and feedback and peer-comparison interventions, has demonstrated reductions in inappropriate antibiotic prescribing in primary care settings [7,8].

Social-norm and behavioral “nudge” interventions, such as accountable justification at the point of prescribing, have similarly demonstrated sustained prescribing improvements across large practice networks [9]. In contrast, stand-alone digital decision support interventions have shown variable effectiveness, often depending on clinician uptake, workflow integration, and contextual fit [10].

Recent pediatric randomized trials further reinforce this heterogeneity. The CHICO cluster-randomized trial evaluated a multifaceted prognostic and communication intervention without demonstrating a significant reduction in antibiotic dispensing, while maintaining non-inferior safety outcomes [11]. A large pragmatic cluster-randomized trial in rural China demonstrated that combined physician and caregiver education significantly reduced antibiotic prescribing for pediatric upper respiratory tract infections [12]. More recently, pediatric-focused outpatient stewardship programs integrating audit, feedback, and communication strategies have been associated with sustained reductions in inappropriate prescribing in routine practice [13]. Nevertheless, uncertainty remains regarding which intervention mechanisms most consistently influence antibiotic prescribing behavior in exclusively pediatric outpatient and primary care populations.

Existing reviews frequently combine adult and pediatric populations, merge inpatient and outpatient settings, or focus on isolated intervention classes, thereby limiting their applicability to pediatric ambulatory care [14]. To our knowledge, no prior review has specifically compared randomized pediatric outpatient antimicrobial stewardship interventions using a mechanism-based framework to evaluate the relative consistency of intervention effects across distinct stewardship strategies.

We therefore conducted a systematic review of randomized controlled trials evaluating outpatient pediatric interventions designed to influence antibiotic prescribing for ARTIs. Our objective was to compare the consistency of effects across predefined antimicrobial stewardship intervention mechanisms and identify which approaches demonstrate the most reliable influence on pediatric outpatient antibiotic prescribing behavior.

## Review

Materials and methods

Protocol and Reporting Standards

This systematic review was conducted in accordance with Preferred Reporting Items for Systematic reviews and Meta-Analyses (PRISMA) 2020 reporting guidelines [15]. Given the substantial clinical and methodological heterogeneity across included trials, a quantitative meta-analysis was not performed. Interventions varied widely in mechanism (educational, behavioral, diagnostic, and digital), outcome definitions were not directly comparable (e.g., prescribing rates, dispensing, and appropriateness), and study designs differed in unit of randomization and analysis (cluster versus individual trials). Pooling such heterogeneous data would require strong assumptions and risk producing misleading summary estimates; therefore, a structured narrative synthesis was considered more appropriate. The review protocol was prospectively registered with the Prospective Register of Systematic Reviews (PROSPERO) database (registration number: CRD420261301412).

Literature Search Strategy

A comprehensive electronic search was conducted in PubMed, Embase, the Cochrane Library, and Google Scholar from database inception to January 2026. Reference lists of included studies and relevant reviews were manually screened to identify additional eligible articles. The search strategy combined controlled vocabulary and free-text terms related to pediatric acute respiratory tract infections, antibiotic prescribing, and antimicrobial stewardship or decision-support interventions. No language restrictions were applied at the search stage, provided sufficient data could be extracted. Grey literature was not systematically searched, as the review focused on randomized controlled trials indexed in major databases. The electronic search identified 146 records, which were processed according to the PRISMA flow diagram. The search strategy was developed using a combination of Medical Subject Headings (MeSH) and free-text terms. Boolean operators (AND, OR) were used to combine concepts. The PubMed search strategy was as follows: (“Respiratory Tract Infections”[MeSH] OR “acute respiratory infection” OR ARTI) AND (“Anti-Bacterial Agents”[MeSH] OR antibiotic OR prescribing) AND (pediatric OR children OR child OR paediatric) AND (“Antimicrobial Stewardship” OR intervention* OR decision support OR feedback OR education OR communication) AND (randomized controlled trial OR cluster randomized trial OR RCT).

Eligibility Criteria

This review included randomized controlled trials, including cluster-randomized designs, that enrolled pediatric populations (≤18 years) or reported extractable pediatric data, presenting with acute respiratory tract infections in primary care or outpatient settings. Eligible interventions were required to target antibiotic prescribing behavior and included educational or stewardship programs, behavioral or shared decision-making strategies, diagnostic-guided approaches, and digital or algorithm-based decision-support tools. Comparators included usual care or lower-intensity interventions. Studies were excluded if they involved adult-only populations without extractable pediatric data, were not randomized, were conducted exclusively in inpatient settings, did not target antibiotic prescribing, or did not report extractable antibiotic prescribing outcomes.

Outcomes

The primary outcome was any measure of antibiotic prescribing or dispensing, including immediate or same-day prescribing, dispensing rates, inappropriate antibiotic treatment, or guideline-concordant prescribing. Secondary outcomes included reconsultation or follow-up visits, hospital admission, symptom resolution, and knowledge- or communication-related outcomes.

Study Selection and Data Extraction

All search results were imported into EndNote 20 (Clarivate Analytics) for deduplication and screening. Two reviewers independently screened titles and abstracts, followed by full-text assessment of potentially eligible studies. Disagreements were resolved through discussion or by a third reviewer. After removal of duplicates (n = 38), 108 records were screened, and 22 full-text articles were assessed for eligibility. Fourteen studies were excluded for predefined reasons, leaving eight randomized controlled trials for qualitative synthesis. Data were extracted independently by two reviewers using a standardized form. Extracted variables included study design, setting, sample size, participant characteristics, intervention and comparator details, outcome definitions, and follow-up duration.

Risk of Bias Assessment

Risk of bias was assessed using the Cochrane Risk of Bias 2 (RoB 2) tool. Two reviewers independently evaluated all domains, and disagreements were resolved through discussion or by a third reviewer.

Results 

Study Selection

**Figure 1:**
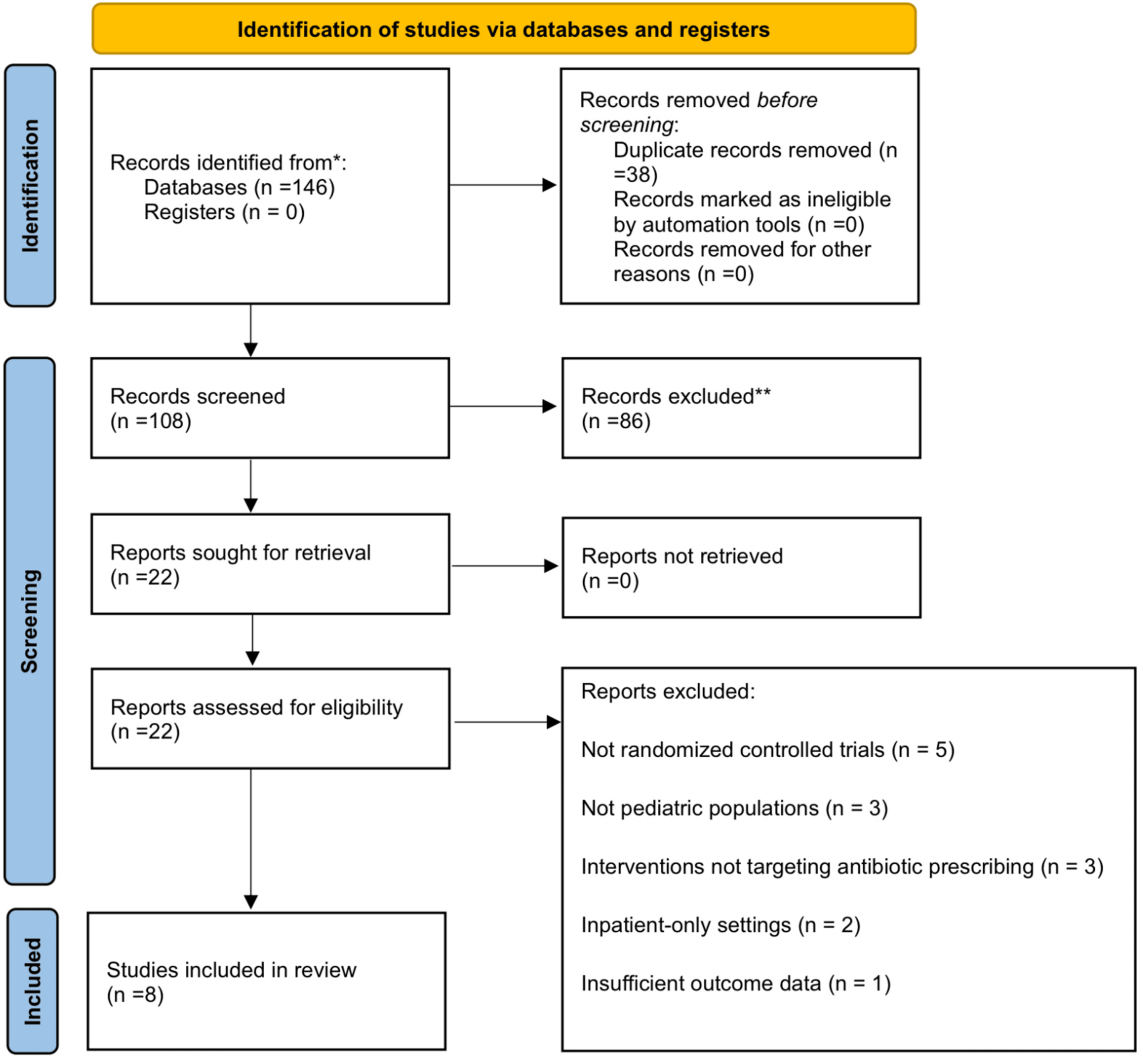
PRISMA 2020 flow diagram of study selection process Preferred Reporting Items for Systematic reviews and Meta-Analyses (PRISMA) 2020 flow diagram illustrating the process of study identification, duplicate removal, screening, eligibility assessment, and inclusion in the qualitative synthesis, adapted from the PRISMA 2020 statement [15].

The systematic search identified 146 records across PubMed, Embase, the Cochrane Library, and Google Scholar. After removing 38 duplicates, 108 unique records remained for title and abstract screening. Following screening, 86 records were excluded for not meeting the eligibility criteria, most commonly because they involved adult populations, non-respiratory conditions, non-randomized designs, or interventions unrelated to antibiotic prescribing. Twenty-two full-text articles were assessed for eligibility. Fourteen were excluded for the following reasons: not randomized controlled trials (n = 5), not pediatric populations (n = 3), interventions not targeting antibiotic prescribing (n = 3), inpatient-only settings (n = 2), and insufficient outcome data (n = 1). Finally, eight randomized controlled trials met the inclusion criteria and were included in the qualitative synthesis. The study selection process is illustrated in Figure 1.

Study Characteristics

**Figure 2:**
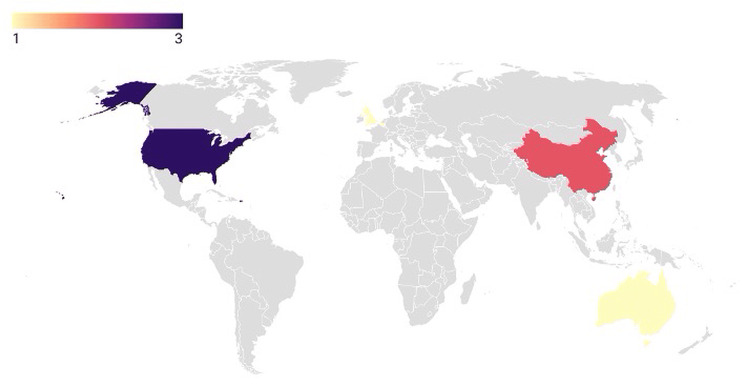
Geographic distribution of included randomized controlled trials Map illustrating the geographic distribution of included randomized controlled trials. Color intensity represents the number of studies conducted in each country. Map created using Datawrapper (https://www.datawrapper.de/).

Eight randomized controlled trials were included. The studies were conducted between 2017 and 2025 across China, Belgium, the United States, Australia, and the United Kingdom. Most trials were undertaken in primary care or general practice settings, while two were performed in pediatric outpatient clinics. All studies focused on ambulatory encounters; none were conducted in inpatient wards. The geographic distribution and key study characteristics are summarized in Figure 2 and Table 1, respectively.

**Table 1:** Characteristics of included randomized controlled trials Summary of study characteristics including country, setting, study design, population, intervention, comparator, and primary outcomes.

Study (year)	Country	Setting	Population (age)	Unit randomized	Study design	Intervention	Comparator	Primary outcome	Main finding
Wei et al. (2017) [12]	China	Primary care township hospitals	2–14 years	Hospitals	Cluster-RCT	Clinician training, guideline dissemination, monthly peer-review, and caregiver education	Usual care	Antibiotic prescription rate	Large absolute reduction in antibiotic prescribing in the intervention group
Lemiengre et al. [7]	Belgium	Primary care	Children with non-severe ARTI	Practices	Factorial cluster-RCT	POC CRP testing, BISNA communication training, or both	Usual care	Immediate antibiotic prescribing	CRP alone showed no reduction; BISNA increased prescribing; combined intervention neutralized the increase
Mann et al. (2020) [10]	United States	Primary care networks	ARTI visits (pediatric data extractable)	Practices	Cluster-RCT	EHR-integrated clinical decision support (iCPR)	Usual care	Overall antibiotic prescribing	No significant difference between groups
Goggin et al. (2022) [16]	United States	Pediatric outpatient clinics	1–5 years	Clinics	Cluster-RCT	Parent–clinician communication training and parent video (high vs low intensity)	Lower-intensity communication	Inappropriate antibiotic treatment	No significant difference between groups
Clegg et al. (2021) [17]	United States	Family medicine clinics	Pediatric patients	Clinics	Cluster-RCT	Monthly individual clinician feedback and education	Clinic-level feedback	Guideline-concordant prescribing	Individual feedback significantly improved prescribing
Hoffmann et al. (2022) [18]	Australia	General practice	ARI patients	Practices	Cluster-RCT	Brief decision aids and GP training	Usual care	Antibiotic dispensing rate	No between-group difference; clinician knowledge improved
Li et al. (2025) [19]	China	Pediatric outpatient department	Children	Patients	Individual RCT	Point-of-care PCR testing	Routine care	Same-day antibiotic prescribing	Relative reduction in antibiotic prescribing
Blair et al. (CHICO trial, 2023) [11]	United Kingdom	General practice	0–9 years	Practices	Cluster-RCT	Prognostic algorithm, concern elicitation, and safety-net leaflet	Usual care	Antibiotic dispensing and hospital admission	No difference in antibiotic dispensing; non-inferior hospital admissions

All trials enrolled children with acute respiratory tract infections or reported extractable pediatric data, predominantly non-severe. Reported age ranges extended from 6 months to 14 years. Several trials recruited general practitioners, family physicians, or pediatricians. Outcomes were derived from pediatric consultations and prescriptions, with analyses conducted at the patient or encounter level while accounting for clustering where applicable. With regard to design, six studies were cluster-randomized controlled trials, one was a factorial cluster-randomized trial, and one was an individually randomized controlled trial. In the cluster trials, randomization occurred at the level of the clinic or practice. Most studies used mixed or hierarchical regression models to account for clustering effects.

The interventions were classified into four categories. Educational and stewardship-based interventions included clinician training, guideline dissemination, peer or individual feedback, and caregiver education. Behavioral and shared decision-making interventions consisted of brief decision aids, communication training, and parent-directed educational materials. Diagnostic-guided interventions used point-of-care tests, including C-reactive protein testing and multiplex polymerase chain reaction assays, to inform prescribing decisions. Digital and algorithm-based interventions comprised electronic clinical decision support systems and prognostic risk algorithms combined with safety-netting materials.

All trials compared the intervention against usual care or a lower-intensity comparator. The primary outcome in every study was a measure of antibiotic prescribing or dispensing (e.g., immediate or same-day prescribing, dispensing rates, inappropriate prescribing, or guideline-concordant prescribing). Secondary outcomes varied and included reconsultation, hospital admission, symptom resolution, and clinician knowledge or communication measures.

Intervention Categories

The included interventions were classified into four categories according to their primary mechanism of action to structure the subsequent narrative synthesis. Educational and stewardship-based interventions targeted prescribing behavior through clinician training, audit or feedback, and caregiver education. Two trials fell into this category: Wei et al. [12], which implemented a multifaceted stewardship program combining clinician training and caregiver education, and Clegg et al. [17], which compared individualized clinician feedback with clinic-level feedback. Behavioral and shared decision-making interventions aimed to modify clinician-parent communication and treatment expectations. Hoffmann et al. [18] evaluated brief decision aids accompanied by general practitioner training, while Goggin et al. [16] assessed communication training supported by a parent-directed educational video.

Diagnostic-guided interventions integrated point-of-care testing into routine assessment to inform prescribing decisions. Lemiengre et al. [7] evaluated C-reactive protein testing, whereas Li et al. [19] assessed rapid multiplex polymerase chain reaction testing in pediatric outpatient settings. Digital and algorithm-based tools relied on computerized or prognostic systems to guide prescribing. Mann et al. [10] implemented an electronic clinical decision support tool within the electronic health record, and Blair et al. [11] (CHICO trial) used a prognostic algorithm combined with safety-netting materials. To enhance interpretability and address heterogeneity across intervention types, interventions were not only categorized by mechanism but also comparatively evaluated for the consistency and direction of their effects on antibiotic prescribing outcomes across trials. This approach allowed identification of patterns in intervention performance beyond descriptive classification, enabling more structured interpretation of heterogeneous evidence.

Diagnostic-Guided Interventions

Two randomized controlled trials evaluated diagnostic-guided strategies designed to inform antibiotic prescribing decisions at the point of care. Lemiengre et al. [7] assessed point-of-care C-reactive protein testing in primary care, while Li et al. [19] evaluated rapid multiplex polymerase chain reaction testing in pediatric outpatient settings. In both studies, antibiotic prescribing at the index consultation or on the same day was the primary outcome. The effects of diagnostic-guided strategies were mixed. Lemiengre et al. [7] found that point-of-care C-reactive protein testing alone did not reduce antibiotic prescribing, and when combined with a communication intervention, it neutralized an otherwise increased prescribing tendency. In contrast, Li et al. [19] reported a modest reduction in same-day antibiotic prescribing in the PCR-guided group compared with routine care. Due to substantial heterogeneity in outcome definitions, intervention mechanisms, and analytical units across studies, quantitative synthesis was not performed (Table 2).

**Table 2:** Diagnostic-guided interventions and antibiotic prescribing outcomes Summary of randomized controlled trials evaluating diagnostic-guided interventions, including study setting, intervention type, comparator, outcome definitions, and main findings.

Study	Country	Setting	Sample size	Intervention	Comparator	Primary outcome definition	Main finding	Notes
Lemiengre et al. [7]	Belgium	Primary care	Factorial cluster RCT (multi-arm)	Point-of-care C-reactive protein (POC-CRP)	Usual care / BISNA	Immediate antibiotic prescribing at index visit	POC-CRP alone did not reduce prescribing; BISNA increased prescribing; combined intervention neutralised the increase	Multi-arm factorial design
Li et al. [19]	China	Pediatric outpatient clinics	368 vs 354	Point-of-care multiplex PCR	Routine care	Same-day antibiotic prescribing	54/368 vs 56/354; significant reduction in PCR group	Intention-to-treat analysis

Educational and Behavioral Strategies

Four trials evaluated educational or communication-based strategies aimed at modifying clinician prescribing behavior. Two studies incorporated stewardship components. Wei et al. implemented a multifaceted program combining clinician training, peer review, and caregiver education and reported a marked reduction in antibiotic prescribing [12]. Clegg et al. compared individualized clinician feedback with clinic-level feedback and demonstrated significantly higher guideline-concordant prescribing in the individualized feedback group [17]. In contrast, two trials based primarily on behavioral and shared decision-making approaches did not demonstrate significant reductions in antibiotic prescribing. Hoffmann et al. tested brief decision aids supported by general practitioner training and found no difference in antibiotic dispensing compared with usual care [18]. Similarly, Goggin et al. evaluated high versus low-intensity parent-clinician communication interventions and observed no between-group difference in inappropriate antibiotic treatment [16]. Collectively, these findings indicate that interventions incorporating accountability mechanisms were more likely to influence prescribing behavior than communication-focused strategies alone. 

Digital and Algorithm-Based Tools

Two trials evaluated digital or algorithm-based interventions designed to guide antibiotic prescribing decisions. Mann et al. [10] implemented an electronic clinical decision support tool integrated within the electronic health record, while Blair et al. [11] (CHICO trial) evaluated a prognostic risk algorithm combined with structured concern elicitation and safety-netting materials in primary care. Neither intervention resulted in a significant reduction in antibiotic prescribing compared with usual care. Mann et al. reported low tool uptake, and the intervention was not associated with changes in prescribing behavior [10]. Similarly, Blair et al. found no difference in antibiotic dispensing, despite the use of a risk-based algorithm [11]. These findings suggest that digital or algorithmic decision-support tools alone may be insufficient to change prescribing behavior without high clinician engagement or complementary stewardship strategies.

Secondary Outcomes

Secondary outcomes related to safety, symptom course, and communication were inconsistently reported across studies. Where assessed, no intervention was associated with an increase in serious adverse events or hospital admissions. Reconsultation or follow-up contact was reported by Goggin et al. [16], Li et al. [19], and Blair et al. [11] (CHICO trial), with no statistically significant between-group differences, indicating that reductions in antibiotic prescribing did not increase subsequent healthcare use. Hospital admission was reported only by Blair et al. as a prespecified safety outcome and demonstrated non-inferiority compared with usual care [11]. Symptom resolution, including fever recovery, was evaluated by Li et al. [19] and showed no significant between-group difference, while this outcome was not reported in the remaining trials. Process-related outcomes differed from clinical outcomes: Hoffmann et al. [18] reported significant improvements in clinician knowledge following decision-aid use, and Goggin et al. [16] observed improvements in communication measures; however, neither translated into reductions in antibiotic prescribing (Table 3).

**Table 3:** Secondary outcomes reported across included trials Summary of reported secondary outcomes including reconsultation, hospital admission, symptom resolution, and knowledge or communication-related outcomes across included studies.

Outcome	Studies reporting	Direction of effect
Reconsultation	Goggin et al. [16]; Li et al. [19]; Blair et al. [11]	No statistically significant between-group differences
Hospitalisation	Blair et al. [11] (CHICO trial)	Non-inferior compared with usual care
Symptom resolution	Li et al. [19]	No statistically significant difference
Knowledge/communication outcomes	Hoffmann et al. [18]; Goggin et al. [16]	Improved process and knowledge measures without a change in prescribing

Risk of Bias

**Figure 3:**
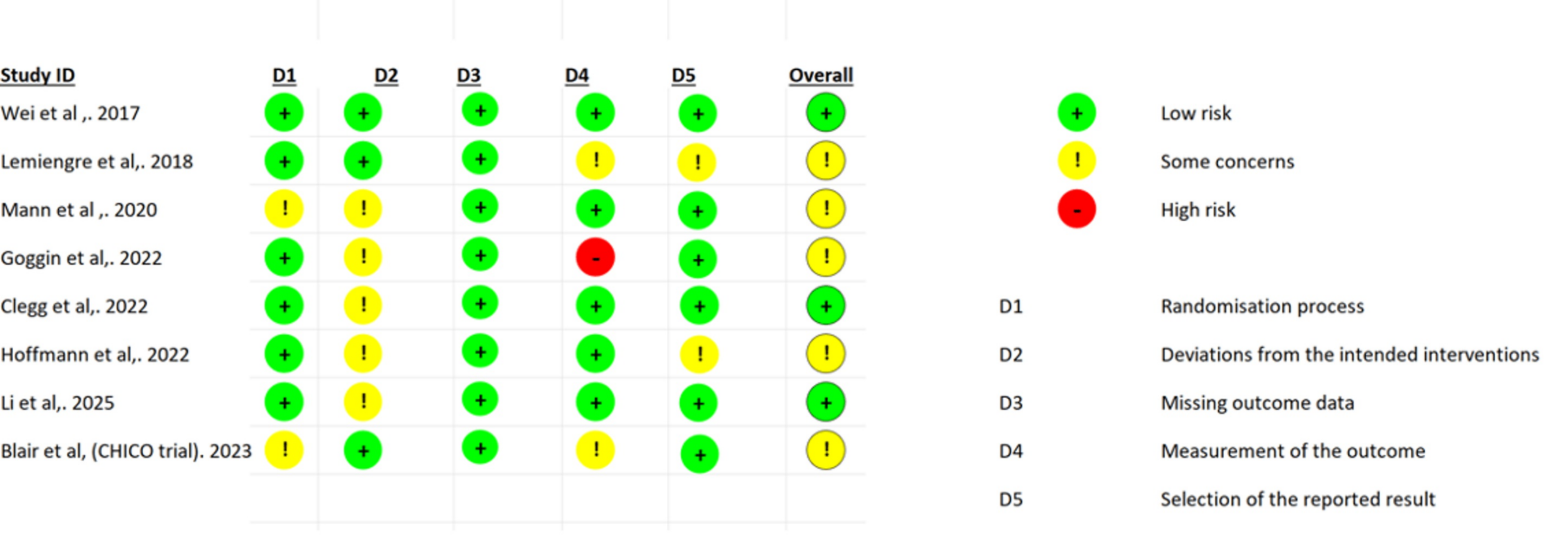
Risk of bias summary (RoB 2) across included randomized controlled trials Studies included: Lemiengre et al. [7], Mann et al. [10], Blair et al. [11], Wei et al. [12], Goggin et al. [16], Clegg et al. [17], Hoffmann et al. [18], Li et al. [19].

The included studies demonstrated predominantly low risk of bias across most domains. Several studies showed consistently low risk across all or nearly all domains, reflecting strong methodological quality [12,17,19]. Some concerns were identified, most commonly in domains related to deviations from intended interventions (D2) and selection of the reported result (D5). Only one study exhibited a high risk in a single domain, which may affect internal validity [16]. Overall, the risk of bias across studies was judged to be low to moderate, as summarized in Figure 3.

Discussion

This systematic review synthesized randomized evidence on outpatient pediatric interventions designed to influence antibiotic prescribing for acute respiratory tract infections (ARTIs). Across the included trials, intervention effects were heterogeneous, with relatively more consistent directional effects observed for strategies incorporating accountability and performance feedback rather than those relying primarily on education, communication tools, or stand-alone decision support. This pattern suggests that differences in intervention mechanisms and implementation context, rather than inconsistency in the evidence base itself, may explain the observed variability in effects [14]. Several sources of heterogeneity merit consideration. First, outcome definitions varied substantially across trials, including immediate or same-day prescribing, overall antibiotic dispensing, and measures of guideline-concordant or inappropriate treatment. These outcomes capture distinct clinical decision points and are not directly interchangeable. Second, most studies employed cluster-randomized designs but differed in unit of allocation and analytic handling of clustering, which can influence effect estimates and precision. Third, baseline prescribing rates likely modified observed effects, as stewardship interventions implemented in settings with relatively low baseline antibiotic use may demonstrate attenuated absolute reductions due to ceiling effects [20].

Despite the diversity of included intervention strategies, a comparative pattern was observed across the included trials, with interventions incorporating accountability and performance feedback showing relatively more consistent directional effects toward reducing inappropriate antibiotic prescribing, whereas communication-based, diagnostic-guided, and stand-alone digital interventions showed variable or neutral effects across studies. This comparative synthesis enables interpretation beyond descriptive categorization and highlights which intervention strategies are more likely to influence prescribing behavior in real-world outpatient pediatric settings. From an implementation perspective, these findings suggest that prioritizing accountability-based and feedback-driven stewardship interventions may yield more reliable improvements in prescribing practices compared with single-component or technology-dependent approaches.

Interpretation of these findings should consider study quality. Most trials were judged to have a low-to-moderate risk of bias, with concerns mainly related to deviations from intended interventions and selective reporting. The observed pattern, particularly the consistent directional effects of accountability-based interventions, remained evident among studies at lower risk of bias, supporting the robustness of the comparative signal despite methodological variability.

Interventions embedding measurement, audit, and feedback demonstrated the most consistent directional effects across the included studies. Such approaches plausibly operate by increasing the salience of prescribing behavior, enabling peer comparison, and introducing accountability into routine clinical practice. Behavioral science frameworks suggest that individualized and credible feedback can shift clinicians from habitual to deliberative decision-making, particularly when integrated into existing workflows with minimal cognitive burden. Similar effects have been reported in recent randomized trials and syntheses of outpatient antibiotic stewardship, supporting the role of accountability-linked strategies in influencing prescribing norms [14,21].

In contrast, interventions centered primarily on shared decision-making, education, or communication aids did not consistently reduce antibiotic prescribing. Although these strategies may improve clinician knowledge, communication quality, or caregiver satisfaction, they may be insufficient to alter the dominant drivers of prescribing in busy outpatient pediatric settings, such as diagnostic uncertainty, time pressure, and clinician risk aversion. In cluster-randomized designs, contamination between intervention and control clinicians through shared practice culture or informal knowledge transfer may further attenuate observable between-group differences. Importantly, the absence of prescribing reductions should not be interpreted as a lack of value, as several trials reported improvements in process-related outcomes without corresponding changes in antibiotic use [22].

Diagnostic-guided interventions demonstrated mixed effects, highlighting the complexity of translating reduced uncertainty into altered prescribing behavior. While point-of-care testing can theoretically support antibiotic restraint by clarifying disease etiology, its effectiveness depends on pretest probability, turnaround time, clinician trust, and alignment with explicit prescribing pathways. Reduced diagnostic uncertainty does not automatically translate into lower antibiotic use if decision thresholds and workflow integration are not aligned. Contemporary stewardship guidance emphasizes that diagnostic tools are most effective when coupled with clear action frameworks and reinforcement strategies rather than deployed as isolated interventions. Similarly, digital and algorithm-based decision support tools alone were not consistently associated with reduced prescribing. Limited clinician uptake, alert fatigue, and workflow friction may undermine their potential impact, particularly in short outpatient encounters. These findings reinforce the distinction between theoretical efficacy and real-world effectiveness. Decision support may be necessary to standardize care, but it is rarely sufficient without complementary accountability mechanisms or organizational incentives. Evidence from implementation research underscores that usability and fidelity often determine success more than the sophistication of the algorithm itself [23].

Safety outcomes are a critical consideration in pediatric antibiotic stewardship. Where reported, reductions or neutral effects on prescribing were not associated with increases in hospital admissions, reconsultations, or symptom deterioration, suggesting that antibiotic restraint did not compromise short-term clinical safety. This observation aligns with growing recognition that unnecessary antibiotic exposure in childhood carries its own risks, including disruption of the gut microbiota and increased susceptibility to adverse effects. Emerging evidence continues to highlight the importance of balancing immediate clinical reassurance against longer-term individual harms associated with antibiotic overuse [3,24].

This review has several limitations. Substantial clinical and methodological heterogeneity limited direct comparability across trials and precluded quantitative synthesis without imposing strong assumptions. Reporting of intervention uptake and implementation fidelity was inconsistent, constraining mechanistic interpretation. Publication bias cannot be excluded, particularly as grey literature was not systematically searched. Nevertheless, the observed pattern favoring accountability-linked interventions over single-component educational or digital strategies suggests a coherent and biologically plausible pattern that is consistent with contemporary outpatient stewardship literature [14,20-23].

## Conclusions

Across randomized trials in pediatric outpatient and primary care settings, intervention effects on antibiotic prescribing were heterogeneous, with a more consistent pattern observed for strategies incorporating measurement, feedback, and accountability. These findings suggest that information provision, communication tools, or decision support alone may be insufficient to change prescribing behavior without reinforcement mechanisms embedded in routine practice. Outpatient pediatric stewardship efforts may be better supported by multicomponent approaches that align behavioral, organizational, and implementation considerations. Future research should standardize outcome definitions and explicitly report intervention uptake and fidelity to guide effective and scalable stewardship strategies.
